# ADOPT model combined with structured health education alleviates the preoperative anxiety of patients undergoing preventive ileostomy

**DOI:** 10.3389/fpsyt.2026.1754253

**Published:** 2026-05-08

**Authors:** Shuwen Wu, Yingting Zhang, Yayun Pan, Jing Qin

**Affiliations:** 1 Department of Anesthesiology, Changhai Hospital, Naval Medical University, Shanghai, China; 2 Department of Gastrointestinal Surgery, Changhai Hospital, Naval Medical University, Shanghai, China

**Keywords:** ADOPT model, ileostomy anxiety, nursing intervention, STAI (state trait anxiety inventory), structured health education

## Abstract

**Objective:**

This study aimed to evaluate the efficacy of the ADOPT (Attitude-Definition-Openmind-Plan-Try it out) model combined with structured health education in alleviating preoperative anxiety in patients undergoing preventive ileostomy for rectal cancer.

**Methods:**

This is a randomized controlled trial. A total of 60 patients scheduled for temporary ileostomy were randomly assigned to either the control group (routine care) or the research group (ADOPT model combined with science popularization interventions). The research group received structured education via a multimedia resource library, including preoperative, intraoperative, and postoperative care guidance, alongside interactive support from a specialized healthcare team. Anxiety levels were assessed with the State-Trait Anxiety Inventory (STAI) at admission and preoperatively.

**Results:**

At baseline, no significant differences were observed in gender (P = 0.202), age (P = 0.052), or BMI (P = 0.798) between the two groups. Both groups exhibited comparable anxiety levels at admission. However, one hour before surgery, the research group showed significantly lower state anxiety (S-AI) scores and total anxiety scores compared to the control group (20 ± 0.48 vs 23 ± 0.37, p<0.001), while trait anxiety (T-AI) scores remained similar (p<0.05).

**Conclusion:**

The integration of the ADOPT model with structured health education effectively reduces preoperative anxiety in ileostomy patients, highlighting its potential as a standardized nursing intervention.

## Introduction

In China, the incidence of rectal cancer ranks second among digestive tract tumors. Surgery is the dominant treatment method for rectal cancer patients, and ileostomy is one of the most common surgical methods for rectal cancer (1, 2). Each year, there are approximately 100,000 new cases of ileostomy patients in China, and the number is growing rapidly. Due to the loss of self-control over defecation and the alteration of defecation in postoperative patients, significant changes have occurred in physical functions and psychological states, seriously affecting their quality of life (3, 4). It can cause them undergoing a strong stress response, among which the most typical symptom is anxiety (5). This psychological stress response not only affects the nervous, endocrine and circulatory systems, but also interferes with the smooth implementation of surgery and anesthesia and affects the prognosis (6). Therefore, how to alleviate the common psychological problems has become a major hotspot in the clinical nursing research of patients with rectal cancer who are undergoing temporary stoma.

Medicine science popularization holds significant importance in clinical practice. It not only helps to prevent the occurrence of diseases but also to enable many diseases that have already occurred to have a better prognosis (7). In this rapidly evolving digital era, medical rumors are often spread infinitely. It is even more necessary for medical staff to convey truly useful and correct popular science knowledge to the public, as to alleviate the common psychological problems of patients (8).

The Attitude-Definition-Openmind-Plan-Try it out (ADOPT) model, with the joint participation of medical staff, patients and their families, is based on five steps to propose and solve the problems existing in clinical practice. A new nursing model that promotes the recovery of patients’ conditions. This study explored the application of the popular science nursing intervention model based on the ADOPT model for preoperative anxiety in patients undergoing temporary stoma.

## Methods

### Patients

Rectal cancer patients who were prepared for a temporary ileostomy at Changhai Hospital from June 2023 to February 2024 were included. The inclusion criteria were as follows: the distance between the tumor and the anal verge was less than 5cm and a temporary ileostomy was planned; no mental disease and history of anxiety or depression; with a certain ability to understand and operate; aging from 18–60 years old; ASA score: I-III; agree and sign the informed consent form. Exclusion criteria were as follows: with psychological problems before the operation; developed severe surgical complications during hospitalization; emergency surgery patients. Finally, 60 patients were screened out and divided into the study group and the control group according to the random number, with 30 cases in each group. This study was approved by the hospital ethics committee.

### Nursing

Routine care was provided to the control group, which specifically includes introducing disease-related knowledge, the admission environment, routine preoperative preparations, key points of intraoperative cooperation, specific measures for preventing complications, and postoperative precautions, etc (9).

As for the research group, on the basis of the control group, the ADOPT model was used in combination with popular science nursing intervention. It includes the establishment of a joint science popularization platform for wards and operating rooms, and the setting up of a temporary structured health education group for stoma patients, consisting of a doctor and 5 nurses (the head nurse of the gastrointestinal surgery department; the head nurse of operating room; a specialist ward nurse, a full-time nurse in the operating room, a Stoma therapist nurse). All members are trained uniformly. Establish a popular science resource library for patients undergoing temporary stoma surgery.

The sources of the library’s content include literature review, clinical experience, patient interview, expert consultation and questionnaire surveys, etc. A series of temporary stoma patient popular science resource libraries have been sorted out, covering “preoperative - intraoperative - postoperative - continuous care - solutions to difficult problems”. Form a popular science resource library via the Internet: Based on the questionnaire survey, a total of 30 questions from patients undergoing temporary stoma surgery were sorted out. Relevant popular science videos were produced and uploaded to the Tencent Video software.

The video library is divided into two parts. The first part is for specialized wards, such as basic knowledge of Stoma, wearing and precautions of stoma pocket. The key steps such as stoma irrigation are presented in the form of animations and pictures, and the key points of care are elaborated in detail. The second part is about the operating room, such as “Introduction to the Operating Room Environment; Introduction to Preoperative preparations; Preparation of the intestinal tract; The surgical process of a temporary stoma; Special instruments for enterostomy” and so on, revealing the operating room in the form of videos. There are a total of 30 videos, including 20 videos about gastrointestinal ward and 10 videos about the operation. The total duration is 60 minutes. Merge all video links into a single QR code, guide patients and their families to scan the code to watch popular science videos based on their own needs, instruct patients to leave messages under the videos, provide real-time answers to patients’ questions, and continuously update the popular science video library.

The specialized ward nurse and the full-time nurse in the operating room respectively strengthened the intervention for patients at two time points: at admission and one day before the operation.

### ADOPT implementation

Attitude (A): When admitted to the hospital, the clinical data of the patient is collected by the nurse in the specialized ward, including basic information, past medical history, treatment experience, medication, lifestyle, nutritional status, and mental condition. And assess the patient’s attitude towards the proposed temporary stoma for rectal cancer through open-ended questions. For example, ask the patient: “How much do you know about your condition?” What’s your opinion on rectal cancer? “Do you know the precautions after stoma surgery?” Guide the patient to express themselves actively, give them timely encouragement and understanding, and relieve their negative emotions.

Definition (D): Define health issues and analyze the health problems existing in the patient, such as lack of understanding of relevant knowledge about rectal cancer, fear and unfamiliarity with surgery, concerns about postoperative quality of life, etc. By considering individual differences of the patients (such as age and educational level), the potential sources of anxiety are analyzed, and targeted solutions are formulated. Before the operation, the specialist nurse in the ward used the resource library to show patients videos such as “Introduction to Preoperative Preparation”, “Basic Knowledge of stoma”, and “Wearing of stoma”, and invited stoma volunteers to share stoma care experiences, such as precautions for changing stoma pockets and postoperative life guidance. Further, the specialist nurse encouraged patients to enhance their confidence in defeating the disease. Before the operation, the specialist nurse in the operating room guided the patient to rehearsing the surgical position and informed the patient through videos about “The process of entering the operating room”, “The room temperature in the operating room”, and “How does anesthesia Begin?” to deepen the patient’s understanding of operation.

Open mind (O): the ward specialist nurse and the operating room specialist nurse respectively encouraged patients to boldly express their inner thoughts and inform them of the medical goals they hoped to achieve at the two time points. The specialist nurse encouraged patients and their families to participate in the formulation of the care plan, express their needs and suggestions, such as details about the surgery they wish to know, key points of postoperative care, etc. Medical staff accepted patient feedback with an open attitude, dynamically adjust intervention strategies, and enhance the patients’ sense of participation.

Plan (P): The structured health education group for temporary stoma patients jointly discusses and formulates a personalized full-cycle care plan based on the actual situation of the patients. Meanwhile, throughout the entire nursing process, patients can offer suggestions on the work of the nursing staff. The temporary stoma patient structured health education group will improve the nursing measures based on the actual situation of the patients and provide timely explanations to them. A multidisciplinary nursing team (including surgeons, nurses, ostomy therapists, etc.) was formed and a personalized care plan covering the entire surgical process was developed.

Try it out (T): Patients can scan the QR code of the resource library to enter the media platforms, where they can leave real-time messages. Members of the temporary stoma patient structured health education group promptly collect patients’ questions and invite experts and professors to answer them in a timely manner. At the same time, they offer encouragement to patients’ psychology and spirit through both language and behavior.

### Evaluation

The STAI scale was developed by Spielberger in 1977 and consists of 40 items. The scale is divided into the state anxiety inventory (S-AI) and the trait anxiety inventory (trait anxiety inventory) (T-AI), each with 20 items, can directly reflect the patient’s anxiety level and can distinguish between current and consistent anxiety conditions. Among them, S-AI is used to evaluate state anxiety under stress, with half of the items being positive and half being negative. TAI is used to assess people’s frequent emotional experiences. There are 11 negative items and 9 positive items. The Likert 4-level scoring method is adopted. Positive items are scored 4 to 1 point in reverse, and negative items are scored 1 to 4 points. The scores of S-AI and T-AI are added together, and the total score ranges from 40 to 160 points. The Cronbach coefficient of this scale was 0.872, indicating good reliability and validity.

### Statistics

Data were statistically processed with SPSS 22.0. Count data were expressed as n (%) and the χ2 test was used. Measurement data were expressed as x ± s, and t test was used. *P value* < 0.05 was considered statistically significant.

## Results

### Patients’ demographic and clinical characteristics at baseline

A total of 60 patients were included in this survey. 120 questionnaires were distributed, and all were filled out by the patients themselves on the spot and promptly retrieved. The recovery rate was 100%. No significant differences were observed in gender (P = 0.202), age (P = 0.052), or BMI (P = 0.798) between the two groups (**Table 1**).

**Table 1:** Clinical and pathological parameters of both groups.

Baseline	Control n=30	Research n=30	*P value*
Age	57.98 ± 12.66	62.95 ± 11.50	0.052*
Gender			
male	19(63.33%)	18(60.00%)	0.202**
female	11(36.67%)	12(40.00%)	
BMI	23.38 ± 3.24	23.54 ± 2.81	0.798*
Tumor height			
<5cm	20(66.67%)	21(70.00%)	0.148**
5-10cm	10(33.33%)	9(30.00%)	
Pathological type			
Squamous carcinoma	7(23.33%)	8(26.67%)	0.752**
adenocarcinoma	23(76.67%)	22(73.33%)	

* denotes P value calculated using the chi squared test; ** denotes P value calculated using the independent samples t-test.

### Anxiety level

At admission, the control group had 19 patients with mild or no anxiety symptoms, 8 with moderate anxiety symptoms, and 3 with severe anxiety symptoms. The research group had 18 patients with mild or no anxiety symptoms, 8 with moderate anxiety symptoms, and 4 with severe anxiety symptoms. When comparing T-AI and S-AI scores between the two groups, the differences were not statistically significant (p > 0.05).

Subsequently, both groups received interventions from our specialized team. One hour before surgery, there was no statistically significant difference in T-AI between the control and research groups. However, a statistically significant difference was observed in S-AI, as well as in the total anxiety score (**Table 2**).

**Table 2:** Comparison of anxiety stage between two groups.

Time	Results	Control (n=30)	Research (n=30)	*P value*
Admission	S-AI	22 ± 1.12	20 ± 0.28	0.372**
	T-AI	25 ± 2.73	24 ± 1.87	0.392**
1h preoperative	S-AI	23 ± 0.37	20 ± 0.48	<0.001**
	T-AI	27 ± 3.29	19 ± 3.90	0.734**

* denotes P value calculated using the chi squared test; ** denotes P value calculated using the independent samples t-test.

## Discussion

The significance of the ADOPT nursing model for clinical work is increasingly important. Wu (10) et al. implemented continuous nursing based on the ADOPT model for gestational diabetes mellitus. The results showed that implementing the ADOPT model for patients with gestational diabetes mellitus could effectively establish communication channels between nurses and patients and help parturients actively participate in the formulation of nursing plans with an open mind. It prompts them to fully realize the importance of blood sugar control, thereby enhancing their enthusiasm and compliance for treatment, and thus improving blood sugar levels, reducing pregnancy risks and the occurrence of adverse effects. This proves the effectiveness of ADOPT in clinical management. Liu (11) et al. implemented personalized treatment based on the ADOPT model for hemodialysis patients. The results showed that it could effectively improve the level of exercise self-ability in maintenance blood patients. In this study, under the personalized care of patients with temporary stoma based on the ADOPT nursing model, an open mindset was fully integrated into the clinical nursing process, effectively establishing a bridge of friendship for communication between nurses and patients. This encouraged patients to boldly express the reasons for their anxiety and fear, thereby further implementing personalized popular science education (12).

With the rapid development in China, the demand for medical knowledge has become more urgent. However, due to the lack of medical knowledge and the influence of false knowledge in publicity, many health and even medical disputes have occurred. Meanwhile, surgery, as a strong stressor causing anxiety among negative life events, has an incidence of anxiety disorders in perioperative patients as high as 40% to 80%. Multiple studies have confirmed that preoperative anxiety will increase the risk of incision infection in patients and prolong the incision healing time (13, 14). At the same time, it causes excitation of the sympathetic nerve in the body, leading to elevated blood pressure and extensive bleeding in the surgical field, which is difficult to stop. How to effectively alleviate this problem has become an urgent matter. Nowadays, medical popular science is one of the important ways to enhance patients’ knowledge of diseases, solve medical puzzles and improve human health levels.

From this study, it can be found that applying the ADOPT model to medical popular science can effectively reduce the preoperative anxiety level of patients. However, at present, the application of popular science in medical management is relatively rare, and a standardized and unified model has not yet been formed. In the future, multi-center studies are still needed to further improve it in order to form a standardized nursing management process.

## Conclusion

The integration of the ADOPT model with structured health education effectively reduces preoperative anxiety in ileostomy patients, highlighting its potential as a standardized nursing intervention. Further multi-center studies are warranted to validate and refine this approach.

## Data Availability

The raw data supporting the conclusions of this article will be made available by the authors, without undue reservation.
